# Supplementary material for: Reconstructing the population history of the sandy beach amphipod Haustorioides japonicus using the calibration of demographic transition (CDT) approach
Source: PLoS One. 2019 Oct 9;14(10):e0223624. doi: 10.1371/journal.pone.0223624 (PMC6785125; doi:10.1371/journal.pone.0223624)
Supplement: S1 Fig — Log-normal and Gaussian approximations of the rate are also shown. (PDF) [file pone.0223624.s001.pdf]

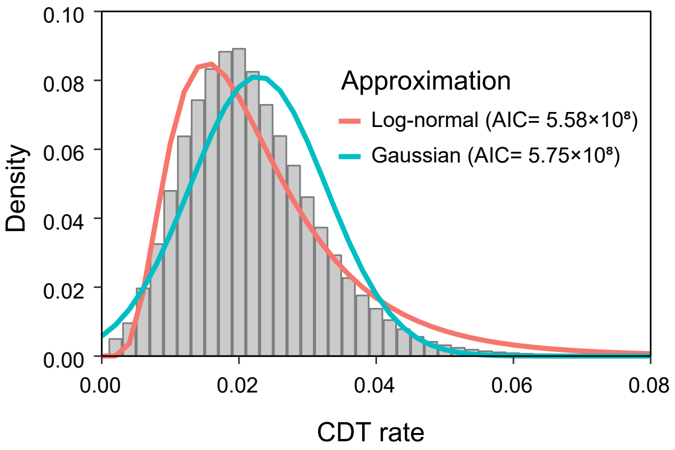

S1 Fig. Histogram showing the density distribution of evolutionary rate derived from CDT.
Log-normal and Gaussian approximation of the rate were also shown.
