# Supplementary material for: Reconstructing the population history of the sandy beach amphipod Haustorioides japonicus using the calibration of demographic transition (CDT) approach
Source: PLoS One. 2019 Oct 9;14(10):e0223624. doi: 10.1371/journal.pone.0223624 (PMC6785125; doi:10.1371/journal.pone.0223624)
Supplement: S1 Table — Haplotype ID, total number of individuals (n), number of individuals per site (Per site), and accession number are shown. (PDF) [file pone.0223624.s002.pdf]

S1 Table. **List of haplotypes.** Haplotype ID, total number of individuals (*n*), number of individuals per site (Per site), and accession number are shown.

ID	*n*	Per site							Accession No.	Reference
		1	2	3	4	5	6	7		
Hja_004	1			1					LC224177	Takada et al., 2017
Hja_006	19	8	2	2	7				LC224179	Takada et al., 2017
Hja_010	35	15	16		1	1	2		LC224183	Takada et al., 2017
Hja_011	1		1						LC224184	Takada et al., 2017
Hja_012	1		1						LC224185	Takada et al., 2017
Hja_013	1	1							LC224186	Takada et al., 2017
Hja_106	1				1				LC474498	This study
Hja_107	1				1				LC474499	This study
Hja_108	1				1				LC474500	This study
Hja_109	2					2			LC474501	This study
Hja_110	1						1		LC474502	This study
Hja_111	12							12	LC474503	This study
Hja_112	1							1	LC474504	This study
Hja_113	1							1	LC474505	This study
Hja_114	1							1	LC474506	This study

Reference

Takada Y, Sakuma K, Fujii T, Kojima S. Phylogeography of the sandy beach amphipod *Haustorioides japonicus* along the Sea of Japan: Paleogeographical signatures of cryptic regional divergences. Estuar Coast Shelf Sci. 2018; 200: 19–30. doi:10.1016/j.ecss.2017.10.012
