# Supplementary material for: Reconstructing the population history of the sandy beach amphipod Haustorioides japonicus using the calibration of demographic transition (CDT) approach
Source: PLoS One. 2019 Oct 9;14(10):e0223624. doi: 10.1371/journal.pone.0223624 (PMC6785125; doi:10.1371/journal.pone.0223624)
Supplement: Attachment — Submitted filename: Response to reviewers.docx [file pone.0223624.s003.docx]

**Response to Reviewer #1**

**Phylogenetic substitution rate used in the comparison.**	The reviewer suggested to use inter- or intraspecific rate for other crustaceans such as 0.7% (Knowlton &Weight 1998) and 1.77% (Copilas-Ciocianu et al. 2019), instead of “general” evolutionary rate of 1% we used in the comparison. It is true that the rate of 1% is not estimated for crustacean species and we have reanalyzed the data with 0.7%/my by Knowlton &Weight (1998). The point in the figure 4 (and 5) is the disparity between general (or easy) rate and CDT rate, and also an uncertainty which is properly expressed by using stochastic clock model. Because showing the results with three (or more) different rates would increase the complexity of the figure (please check attached file Fig3-2.pdf), we would like to show BSPs with two rates. Ultimately a choice of the clock is arbitrary process and it is out of the focus.

Line 41: The statement has changed following the reviewer’s comment.

Line 153: We performed R2 test according to the reviewer’s suggestion and have added the statement regarding the analysis in Materials and methods (Line 153) and Results (Line 220 and Table 2) sections.

Line 159: We have added the statement that “(Hereafter ‘rate’ means substitution rate, not ‘divergence’ rate)” following the reviewer’s suggestion that it should be clarified what the rate means (substitution rate or divergence rate).

Line 160: As the reviewer suggested, “TEMs were built using all individuals from the NWP populations” is just what we mean. We however do not know to which of the five populations the Tohoku population assigned at this point. The statement has therefore been rephrased as follows: “we built TEMs using all individuals from Tohoku as well as its parental population among currently known sandy beach amphipod populations.”.

Lines 163, 231, 243: We have included the result for the strict clocks following the reviewer’s comment, while the result for the exponential TEM with strict clock model is not shown because the run did not converge.

Line 169: “9000 mutation values” has been replaced with “9000 values of the mutation-scaled transition time” for clarity.

Lines 175: We used ‘fitdistr’ function in the MASS v7.3 package, and we have included the name of the package in Line 175.

Line 99, 179, 252: Following the reviewer’s comment, ”BSP” in Line 99 has been spelled out as “Bayesian Skyline Plot” and reference added. Then the statement in Line 179 has been rephrased as follows: “We inferred the demographic history of the Tohoku and its parental population using a BSP.” For the clock model used in the BSP analysis, we stated that “The molecular rate was modeled based on the statistical distribution from CDT.” in Line 183, because the model is not determined at this point. For better clarity, we have replaced the phrase with “The molecular rate was modeled following the best clock model and statistical distribution from CDT”, also added the statement in Result section (Line 255) to show that exponential relaxed clock was applied to BSP.

Line 340: We have added the references for the faster evolutionary rate of crustacean species according to the reviewer’s suggestion. The point is the rate variation among crustacean taxa and we do not discuss each rate here and just introduced them as examples.

**Response to Reviewer #2**

**Scientific name of the sandy beach amphipod.** As suggested by the reviewer, generic name of ‘*Eohaustorioides*’ is also known for a single species of *Haustorioides japonicus* Kamihira 1977, although it seems not to be widely accepted. The genus *Euhaustrioides* was originally introduced in a book chapter without detailed description (Bousfield and Tzvetkova 1982), and the record of WoRMS is solely based on this information. Later Jo (1988) and Kamihira (1999), the authority of this amphipod group, refuted the establishment of *Eohaustorioides*, and the status of the genus seems still controversial. Our research team is now preparing a taxonomic paper with additional refutation on using this name. Furthermore, WoRMS is a web database and has nothing to do with scientific justification, while it argues that the database is controlled by “taxonomic experts”. We therefore avoided using *Eohaustorioides* in this manuscript in the first round. If it is further required to use *Eohaustorioides* in this manuscript, we would like to follow the editor/reviewer’s suggestion.

Line 86: We have corrected the statement following the reviewer’s suggestion.

Line 95: We have rephrased the statement as “genetically distinct from other known populations” following the reviewer’s comment.

Line 97, 98: “BSP” has been spelled out as “Bayesian Skyline Plot”, and the phrase “of the sandy amphipod *Haustorioides japonicus*” has been added after “genealogy”, according to the reviewer’s suggestion.

Line 111: “two sites” has been replaced with “one site”.

Line 116: “a” is inserted after “using” as suggested.

Line 142: GenBank accession numbers are added following the reviewer’s recommendation.

Line 143: Number of sequences are corrected.

Line 216: We have added “Fig 1” after “site 7” following the suggestion.

Lines 219, 223: The phrase “two populations” was replaced with “two group of populations”.

Line 303: The authority is added after the name of the species.

Lines 428, 434, 488: Formatting mistakes in the Reference section has been corrected according to the reviewer’s comments.

Figure 1 has been revised following the reviewer’s comment.

S1 Table and Figure 2: Haplotype Hja_033 has been removed from the figure because it is haplotype from the different genetic population (SJN, northern Sea of Japan) and not included in the analysis. The caption in the Table has been revised to show that “(n)” means total number of individuals.

Table 1 (Line 129): “(*n*)” is included after “sample size”.
